# An ethylene response factor (*MxERF4*) functions as a repressor of Fe acquisition in *Malus xiaojinensis*

**DOI:** 10.1038/s41598-018-19518-4

**Published:** 2018-01-18

**Authors:** Wei Liu, Ting Wu, Qiwei Li, Xinzhong Zhang, Xuefeng Xu, Tianhong Li, Zhenhai Han, Yi Wang

**Affiliations:** 1 Institute for Horticultural Plants, China Agricultural University, Beijing, 100193 P. R. China; 2 Key Laboratory of Physiology and Molecular Biology of Tree Fruit of Beijing, China Agricultural University, Beijing, 100193 P. R. China

## Abstract

Iron (Fe) is an essential element for plants; however, its availability is limited as it forms insoluble complexes in the soil. Consequently, plants have developed mechanisms to adapt to low Fe conditions. We demonstrate that ethylene is involved in Fe deficiency-induced physiological responses in *Malus xiaojinensis*, and describe the identification of MxERF4 as a protein-protein interaction partner with the MxFIT transcription factor, which is involved in the iron deficiency response. Furthermore, we demonstrate that MxERF4 acts as an MxFIT interaction partner to suppresses the expression of the Fe transporter *MxIRT1*, by binding directly to its promoter, requiring the EAR motif of the MxERF4 protein. Suppression of *MxERF4* expression in *M. xiaojinensis*, using virus induced gene silencing resulted in an increase in *MxIRT1* expression. Taken together, the results suggest a repression mechanism, where ethylene initiates the Fe deficiency response, and the response is then dampened, which may require a transient inhibition of Fe acquisition via the action of *MxERF4*.

## Introduction

Iron (Fe) is a critical element for a number of metalloenzymes involved in photosynthesis and respiration, and so imbalances in Fe levels can profoundly affect cellular metabolism. Plants have developed two Fe uptake strategies, Strategies I (non-graminaceous plants) and II (graminaceous plants)^1,2^, respectively. The Strategy I response includes two main processes: i) the ferric chelates reduction at the root surface; and ii) the absorption of the ferrous Fe across the root plasma membrane^3^. The Strategy II response relies on the mugineic acids (Mas) biosynthesis and secretion, which are Fe(III)-solubilizing molecules that take up chelated Fe, and so is referred to as the Chelation Strategy^1^.

It has been demonstrated that plant hormones are involved in signaling associated with the Fe deficiency responses, inducing adaptive responses^4,5^. As an example, ethylene synthesis is induced during Fe starvation in roots, and may be involved in transducing the Fe deficiency signal to induce adaptive changes^6,7^. Indeed, exogenous application of the ethylene precursor, was reported to mimic iron deficiency induced morphological responses in tomato (*Solanum lycopersicum*)^8,9^ and pea (*Pisum sativum*). Elevated ethylene production, which has been observed as a consequence with Fe deficiency, has been further confirmed by studies showing the upregulation of ethylene synthesis related genes, such as *SAMS*, *ACS*, and *ACO*, under such conditions^10–13^. In addition to ethylene synthesis related genes, ethylene signaling genes also show altered expression during Fe starvation^10,14,15^. Notably, FIT (FER-LIKE IRON DEFICIENCY INDUCED TRANSCRIPTION FACTOR) acts a central role in up-regulating the expression of key genes involved in Fe uptake^16–21^. FIT expression is upregulated by Fe deficiency^17,18^ and in response to ethylene^10,22,23^. In addition, EIN3 (ETHYLENE-INSENSITIVE3) and EIL1 (EIN3-LIKE1) can interact with FIT^23^ directly, and the resulting complex also interacts with the Mediator subunits (MED16 and MED25), which positively regulate iron homeostasis^23–25^. FIT may also interact with gene repressors^21^. The relative proportions of active and inactive FIT pools may be affected by differential FIT protein-protein interactions^21^. Recently, a transcription factor ZAT12 was identified which served as a negative regulator of Fe acquisition^21^.

For keeping the cellular ethylene homeostasis and avoiding toxic effect, it has not yet been determined whether there is repressors function as a negative regulator which can modulate ethylene under prolonged Fe deficient conditions. It is possible that ethylene sensitivity is promoted at the earlier stages of Fe deficiency and then slows the ethylene response once it has been initiated via a dampening mechanism. ERFs (Ethylene-responsive element binding factors), also known as EREBPs (ethylene-responsive element binding proteins), that interact with the GCC-box sequence^26^. Most of the ERFs were identified as transcriptional activators^27–33^. Recent studies indicate that some ERF proteins can act as transcriptional repressors, in tobacco and *Arabidopsis*, ERF3 and ERF4 were shown to repress the expression of a GCC-box-containing reporter gene^27,28^. ERF factors also play a role in a variety of developmental processes^34^, abiotic and biotic stress responses^27,35,36^.

To better understand this ethylene regulatory system in iron stress responses, previously we used *Malus xiaojinensis*, a woody plant that is used as a rootstock for apple trees and that exhibits highly efficient Fe uptake^37–39^. Our previous reciprocal grafting experiments showed that iron deficiency responses induction only occurs when *M. xiaojinensis* was used as the rootstock, compared with the iron inefficiency rootstock^38^. Here, we identified a new protein-protein interaction partner of *M. xiaojinensis* FIT, the ethylene response factor (ERF), MxERF4. We show that MxERF4 serves as a repressor of Fe uptake, thereby acting as a regulator in a signal dampening mechanism.

## Results

### Ethylene is involved in Fe deficiency-induced physiological responses

**Figure 1:**
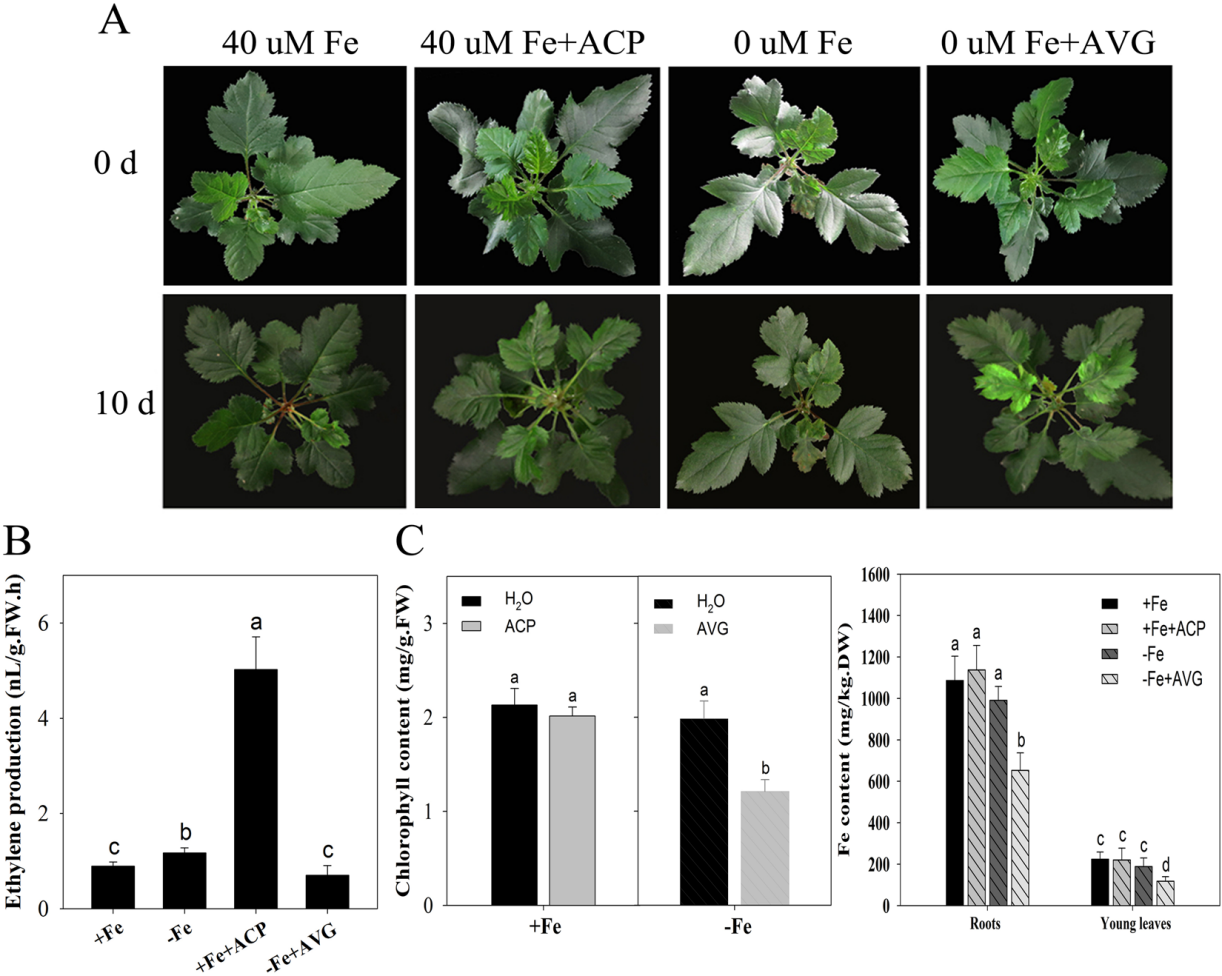
Effect of ethylene on the phenotype of Fe-deficient *Malus xiaojinensis*. (**A**) The phenotype of *M*. *xiaojinensis* seedlings grown in media supplied with 40 µM Fe, 40 µM Fe + 100 mg/L 2-Chloroethylphosphonic acid (ACP), 0 µM Fe, 0 uM Fe + 10 µM aminoethoxyvinylglycine (AVG) for 10 d respectively. (**B**) The ethylene production of *M. xiaojinensis* seedlings grown in media supplied with 40 µM Fe, 40 µM Fe + 100 mg/L ACP, 0 µM Fe, 0 µM Fe + 10 µM AVG for 2 d. Bars represent means ± SE of three replicates. Different letters represent statistically different means at P < 0.05 (one-way ANOVA analysis with Duncan post-hoc test). **(C)** The chlorophyll content and Fe content of *M. xiaojinensis* seedlings grown in media supplied with 40 µM Fe, 40 µM Fe + 100 mg/L ACP, 0 µM Fe, 0 µM Fe + 10 µM AVG for 2 d. Bars represent means ± SE of of three replicates. Different letters represent statistically different means at P < 0.05 (one-way ANOVA analysis with Duncan post-hoc test).

**Figure 2:**
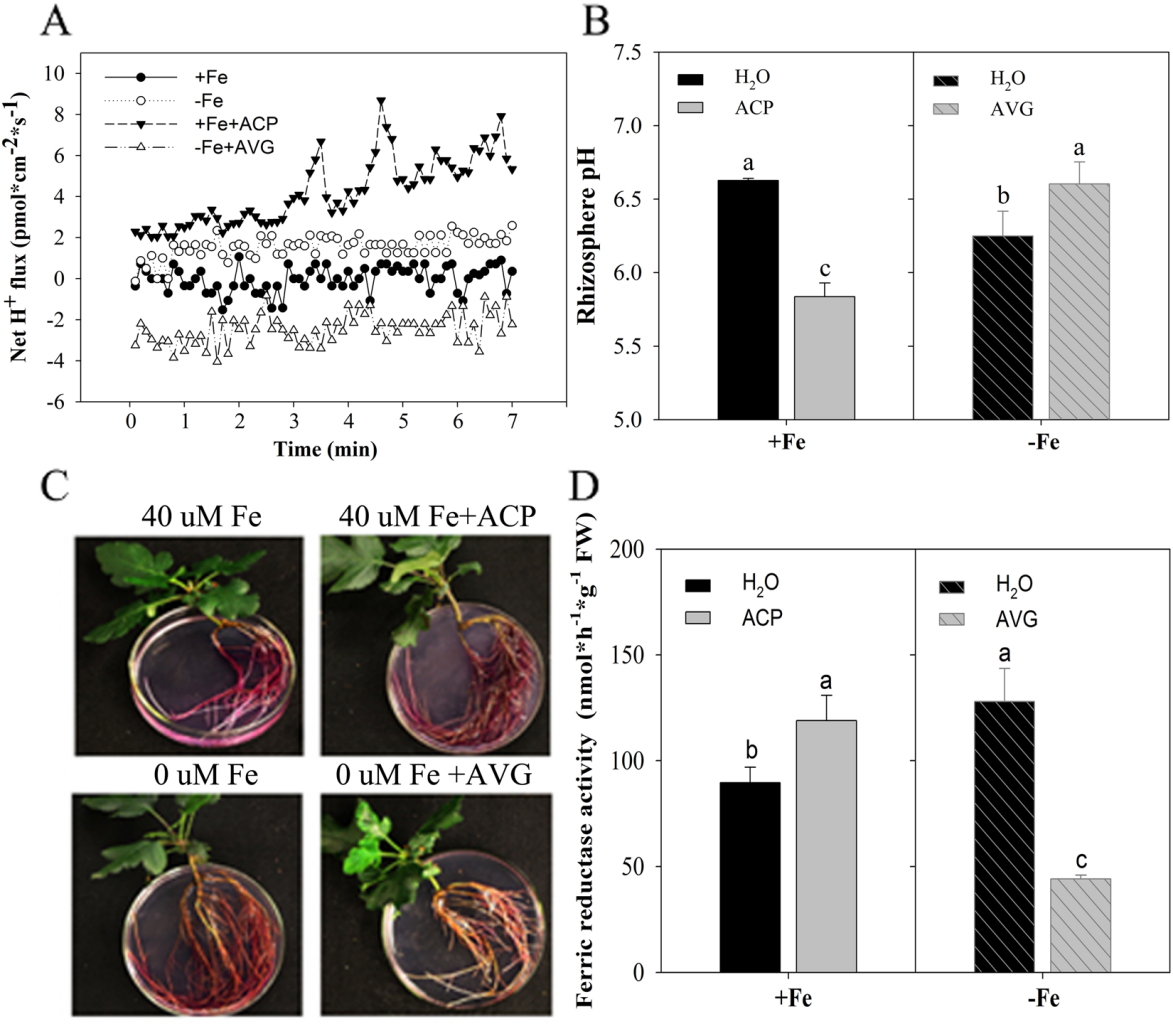
Applying Ethylene treatments effect on Fe-deficient responses in *Malus xiaojinensis*. **(A)** Transient H^+^ flux in the plant root hair zone during different treatments (40 µM Fe, 40 µM Fe + 100 mg/L ACP, 0 µM Fe, 0 µM Fe + 10 µM AVG for 2 d. **(B)** Rhizosphere pH of plants grown under different treatments (40 µM Fe, 40 µM Fe + 100 mg/L ACP, 0 µM Fe, 0 uM Fe + 10 µM AVG) for 2 d. Bars represent means ± SE of three replicates. Different letters represent statistically different means at P < 0.05 (one-way ANOVA analysis with Duncan post-hoc test). **(C,D)** Rhizosphere ferric reductase activity of plants grown under different treatments (40 µM Fe, 40 µM Fe + 100 mg/L ACP, 0 µM Fe, 0 µM Fe + 10 µM AVG) for 2 d. Bars represent means ± SE of three replicates. Different letters represent statistically different means at P < 0.05 (one-way ANOVA analysis with Duncan post-hoc test).

After low Fe treatment of the Fe uptake efficient species, *M. xiaojinensis*s, chlorosis, a typical Fe-deficiency symptom, was not obvious (Fig. 1A). Figure 1B showed Fe deficiency induced the ethylene production and exogenous application of an ethylene inhibitor, aminoethoxyvinylglycine (AVG) decreased ethylene production. Accordingly, AVG application resulted in Fe deficient chlorosis and reduced chlorophyll content under Fe deficient conditions (Fig. 1A,C). Similarly, exogenous application of AVG induced a significant decrease in the Fe content of roots and young leaves but not significant in ACP treatment (Fig. 1C), indicating that ethylene may play a role in the tolerance of Fe deficiency in *M. xiaojinensis*. The regulation of root proton (H^+^) extrusion and Fe(III) reductase (FCR) activity is known to be an important component of the response to Fe deficiency^40–43^, so we assessed whether the Fe deficiency response *M. xiaojinensis* involving ethylene was associated with H^+^ flux and FCR activity. As shown in Fig. 2, Fe deficiency induced a substantial increase in H^+^ flux and FCR activity, but application of AVG weakened this response. Interestingly, application of ethephon, 2-Chloroethylphosphonic acid (ACP) mimicked Fe deficiency in that it induced both increased H^+^ flux and FCR activity under normal Fe conditions. These results suggest that ethylene positively regulates Fe deficiency induced physiological responses in *M. xiaojinensis*.

### Inverse expression of *MxERF4* and Fe deficiency responsive genes during Fe deficiency

**Figure 3:**
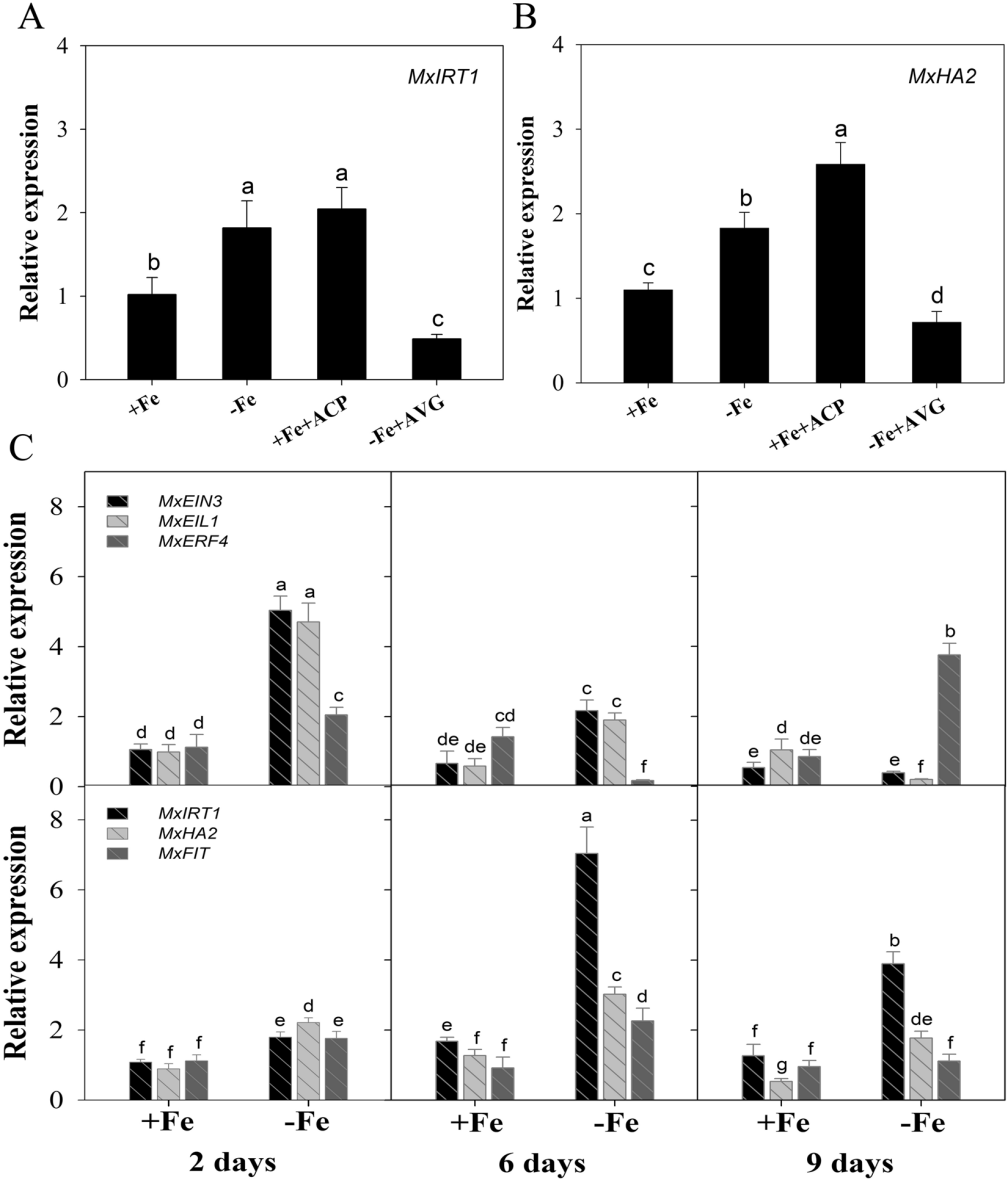
Expression of Fe deficiency responsive genes during ethylene treatments. (**A**) Expression of the *MxIRT1* gene in plants grown under different conditions (40 µM Fe, 40 µM Fe + 100 mg/L ACP, 0 µM Fe, 0 µM Fe + 10 µM AVG) for 2 d. Values are the means ± SE of three replicates. Different letters represent statistically different means at P < 0.05 (one-way ANOVA analysis with Duncan post-hoc test). (**B**) Expression of the *MxHA2* gene in plants grown under different conditions (40 µM Fe, 40 µM Fe + 100 mg/L ACP, 0 µM Fe, 0 µM Fe + 10 µM AVG) for 2 d. Different letters represent statistically different means at P < 0.05 (one-way ANOVA analysis with Duncan post-hoc test). **(C)** Expression of the genes (*MxEIN3*, *MxEIL1*, *MxERF4*, *MxIRT1*, *MxHA2* and *MxFIT*) in plants grown under different conditions (40 µM Fe and 0 µM Fe) for 2 d, 6 d and 9 d. Values are the means ± SE of three replicates.

We first investigated whether Fe deficiency responsive genes were regulated by ethylene application. ACP was included during the normal Fe level treatments and AVG was added when the plants were treated to induce deficiency. We found that the expression levels of *MxHA2* which is responsible for the major acidification activity were increase by the ethylene treatment (Fig. 3A,B). To identify genes potentially involved in ethylene signaling that might control these Fe deficient responsive genes, we queried transcriptome data that was generated from an earlier study of *M. xiaojinensis* exposed to Fe-deficient media^44^. We identified MDP0000324718 as a candidate hub gene for modulating the deficiency response (Fig. S1), which we named *MxERF4*. Expression of *MxERF4* significantly increased in response to Fe deficiency, compared with Fe sufficiency on the ninth day. To determine which biological activities might be coordinated with *MxERF4* expression, we targeted genes that positively regulate Fe homeostasis, and that the expression patterns of *MxFIT*, *MxIRT1* and *MxHA2* were opposite to that of *MxERF4*. As expected, upon Fe deficiency, *MxFIT*, *MxIRT1* and *MxHA2* expression levels were induced during the first 6 days, but were lower at day 9. EIN3 and EIL1, two transcription factors involved in ethylene signaling, have been associated with regulation of iron homeostasis^24^ and, as expected, the expression of both genes was induced during the first 6 days of treatment, but was down-regulated at day 9 (Fig. 3C). Taken together, these results indicated that *MxERF4* may play a role with *MxEIN3* and *MxEIL1* together in the dampening mechanism.

### MxERF4 acts as a repressor and contains a repression domain

**Figure 4:**
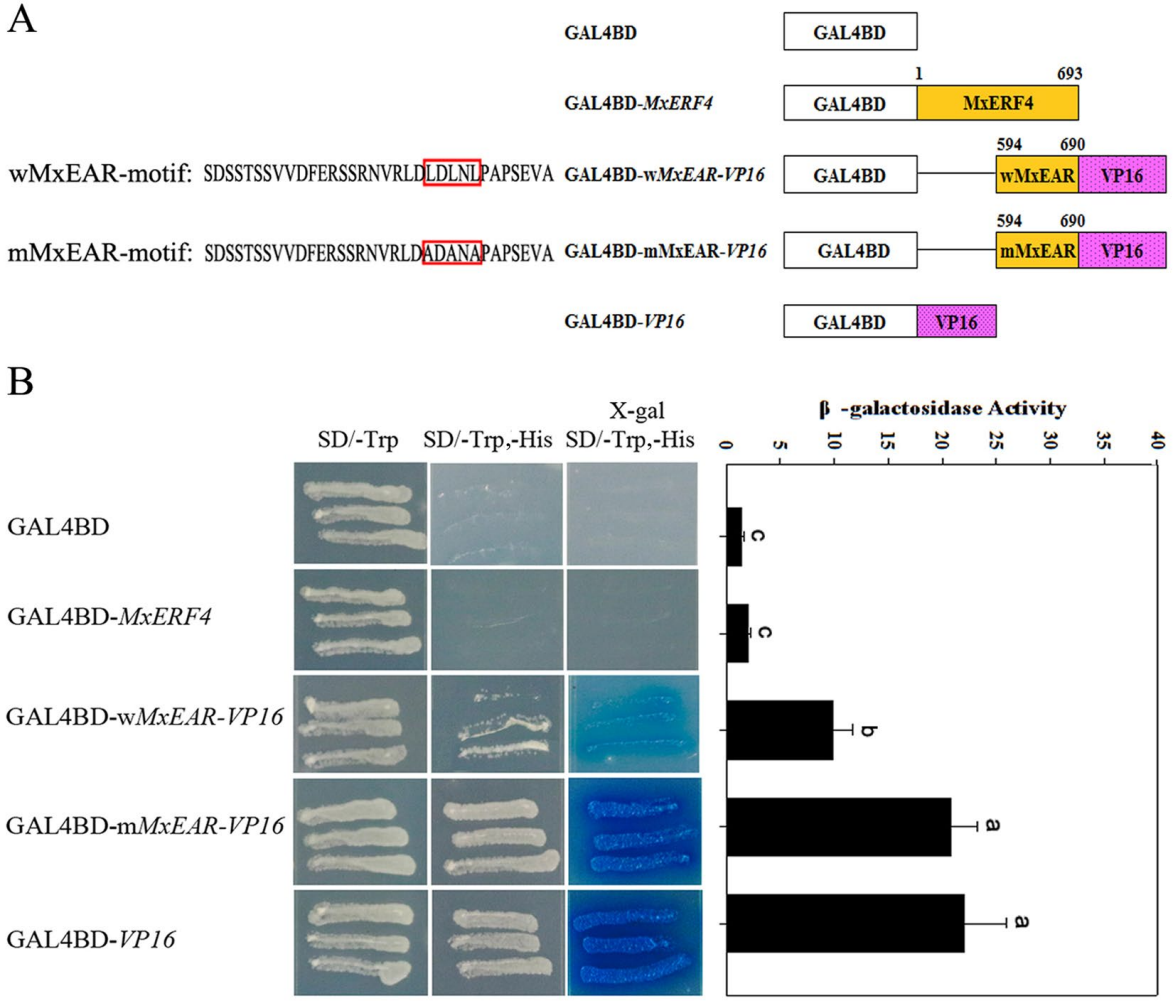
The MxERF4 EAR repression domain inhibits transcriptional activity. (**A**) The EAR-motif point mutation structure diagram of MxERF4 wEAR-motif (LDLNL) mutated as mEAR-motif (ADANA). **(B)** Analysis of X-gal assay of MxERF4 in yeast to identify transactivation activity and β-galactosidase activity. Growth status of yeast AH109 harboring MxERF4 full length (GAL4BD-*MxERF4*), carrying MxERF4 EAR motif of VP16 (GAL4BD-*wMxEAR-VP16*), MxERF4 EAR motif point mutation of VP16 (GAL4BD-*mMxEAR-VP16*) and VP16 (GAL4BD*-VP16*) on minimal medium/-Trp and minimal medium/-Trp,-His, pBD-GAL4 Cam vector (GAL4BD) as control; Transactivation activity by an X-gal assay on minimal medium/-Trp,-His. Values are the means ± SE (n = 9).

Some ERF-associated amphiphilic repression (EAR) motif containing ERF proteins can act as transcriptional repressors. It has been demonstrated that some ERFs can repress gene expression^27,28^; however, the repressor activity of MxERF4 proteins containing EAR motifs need further to be demonstrated. To evaluate potential MxERF4 repressor function, we performed a transactivaiton analysis. We used the VP16 transcriptional activation domain as a positive control and a fusion protein of the EAR motif with VP16 at the N-terminus as the effector (Fig. 4). The results showed that inclusion of the EAR motif with the VP16 resulted in a >50% inhibition of the original β-gal activity (Fig. 4). By contrast, mutation of EAR motif did not affect the activity of the β-gal, indicating that MxERF4 can indeed function as a repressor.

### MxERF4 participates the FIT interaction network and suppresses *MxIRT1* expression

**Figure 5:**
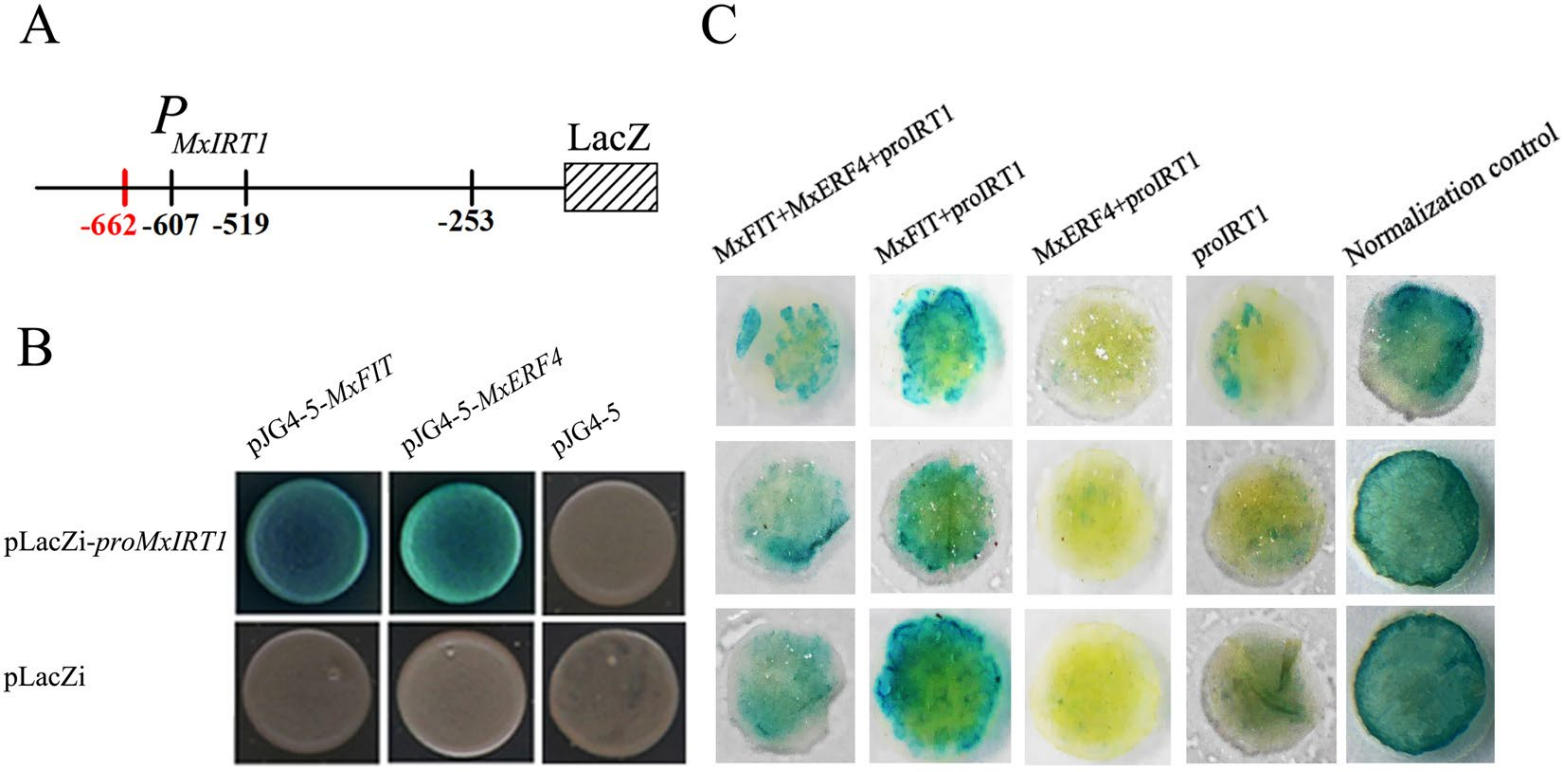
MxERF4 bind the promoter of *MxIRT1* and suppress the effect of MxFIT on *MxIRT1*. **(A)** The structure of the *MxIRT1* promoter (765 bp) showing three GCC-box motifs (−253 bp, −519 bp and −607 bp, in black), one G-box (−662 bp, in red). **(B)** Yeast one hybrid analysis of MxERF4 and MxFIT with the *MxIRT1* promoter. All tests were conducted on minimal medium/-Trp,-Ura. Interactions were determined based on cell growth and were confirmed by an X-gal assay on minimal medium/-Trp,-Ura. **(C)** The effect of MxFIT and MxERF4 on *MxIRT1* promoter. GUS staining of reprentative leaf pieces infiltrated with normalization control (pCAMBIA1301) or only promoter (*MxIRT1*) or coinfiltrated with the transcriptional factor and the promoter (MxFIT + MxERF4 + *proMxIRT1*, MxFIT + *proMxIRT1*, MxERF4 + *proMxIRT1*).

**Figure 6:**
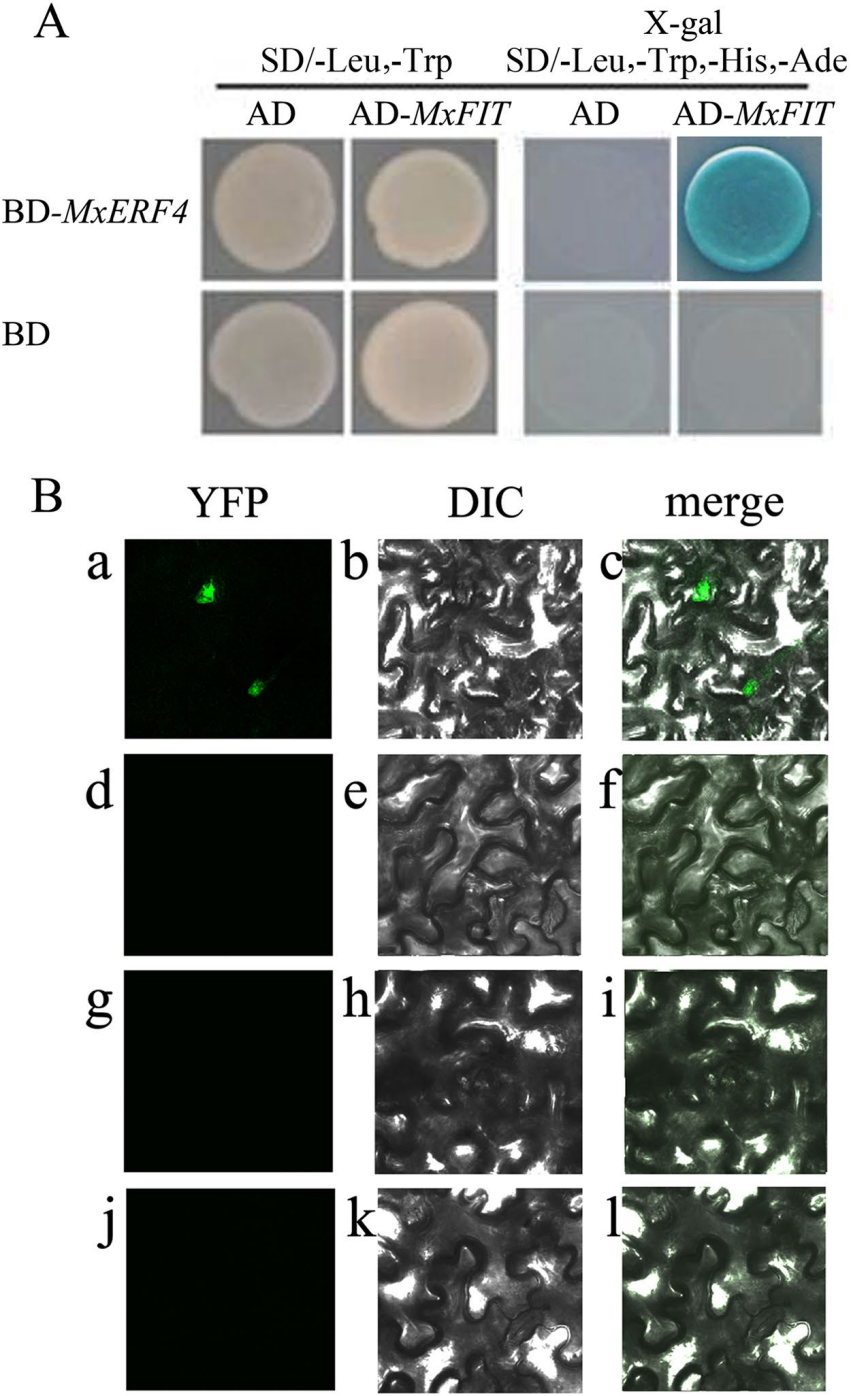
There is an interaction between MxERF4 and MxFIT. (**A**) Yeast two hybrid analysis of the physical interaction between the MxERF4 and MxFIT proteins. The protein interaction was examined using various combinations of prey and bait vectors. All tests were conducted on minimal medium/-Leu,-Trp or on minimal medium/-Leu,-Trp,-His,-Ade. Interactions were determined based on cell growth and were confirmed by an X-gal assay on minimal medium/-Leu,-Trp,-His,-Ade. (**B**) In *planta* protein interaction of MxERF4 and MxFIT. bimolecular fluorescence complementation (BiFC) of yellow fluorescent protein (YFP) in transiently transformed tobacco leaf epidermal cells. The left column ([a], [d], [g] and [j]) shows the YFP signal detected by confocal microscopy; the middle column ([b], [e], [h], and [k]) shows differential interference contrast (DIC) microscopy; the right column ([c], [f], [i], and [l]) shows merge images of the fluorescent signal and DIC. YC-MxFIT plus YN-MxERF4 ([a] to [c]); negative control YC plus YN-MxERF4 ([d] to [f]); YC-MxFIT plus negative control YN ([g] to [i]); negative control YC plus negative control YN ([j] to [l]).

Given that the expression pattern of *MxIRT1* was opposite to that of *MxERF4*, we hypothesized that MxERF4 might serve as a suppressor of *MxIRT1* by binding to its promoter. In support of this idea, the results of a yeast one-hybrid (Y1H) assay (Fig. 5A,B) suggested that both MxERF4 and MxFIT bound to the promoter of *MxIRT1* (*ProIRT1*). Then We investigated the regulation of the *MxIRT1* promoter by MxERF4 and MxFIT using a β-glucuronidase (GUS) transactivation assay in wild tobacco (*Nicotiana benthamiana*) leaves involving co-transformation with the *Pro35S: MxERF4*/*Pro MxIRT1:GUS* or *Pro35S: MxFIT*/*Pro MxIRT1:GUS* constructs. Compared with the pCAMBIA1301 control, when *Pro35S: MxFIT* was co-transformed with *MxIRT1:GUS*, *MxIRT1* promoter activity increased, while MxERF4 decreased *MxIRT1* promoter activity (Fig. 5C). We further hypothesized that since the expression pattern of *MxFIT* was opposite to that of *MxERF4*, the interaction between MxFIT and MxERF4 might be responsible for repression of FIT function. To test for an interaction between MxFIT and MxERF4, we used a targeted yeast two-hybrid (Y2H) assay with the full-length MxFIT open reading frame cloned into the activation domain (AD), named MxFIT-pGADT7, and the MxERF4 coding sequence cloned into the pGBKT7 (BD) vector which harbored the binding domain. We observed an interaction between MxFIT and MxERF4 (Fig. 6A), but no interactions were apparent using BD-MxERF4 together with an empty AD vector, or an empty BD vector with AD-MxFIT, which served as negative controls. To test whether an interaction between MxERF4 and MxFIT would take place in plant cells, we performed an in *planta* bimolecular fluorescence complementation (BiFC) analysis^45^. Following transient transformation of *Nicotiana benthamiana* leaves, yellow fluorescent protein (YFP) signal was detected in the nuclei of leaves expressing YFP-MxFIT and YFP-MxERF4 (Fig. 6B), showing that there is interaction between MxERF4 and MxFIT. Further, by using transient transformation we found that the interaction of MxERF4/MxFIT with the *MxIRT1* promoter inhibited the activity of GUS, respectively, compared with that in the MxFIT with the *MxIRT1* promoter, indicating that MxERF4 acts as an MxFIT interaction partner to serve as a suppressor of *MxIRT1* (Fig. 5C).

**Figure 7:**
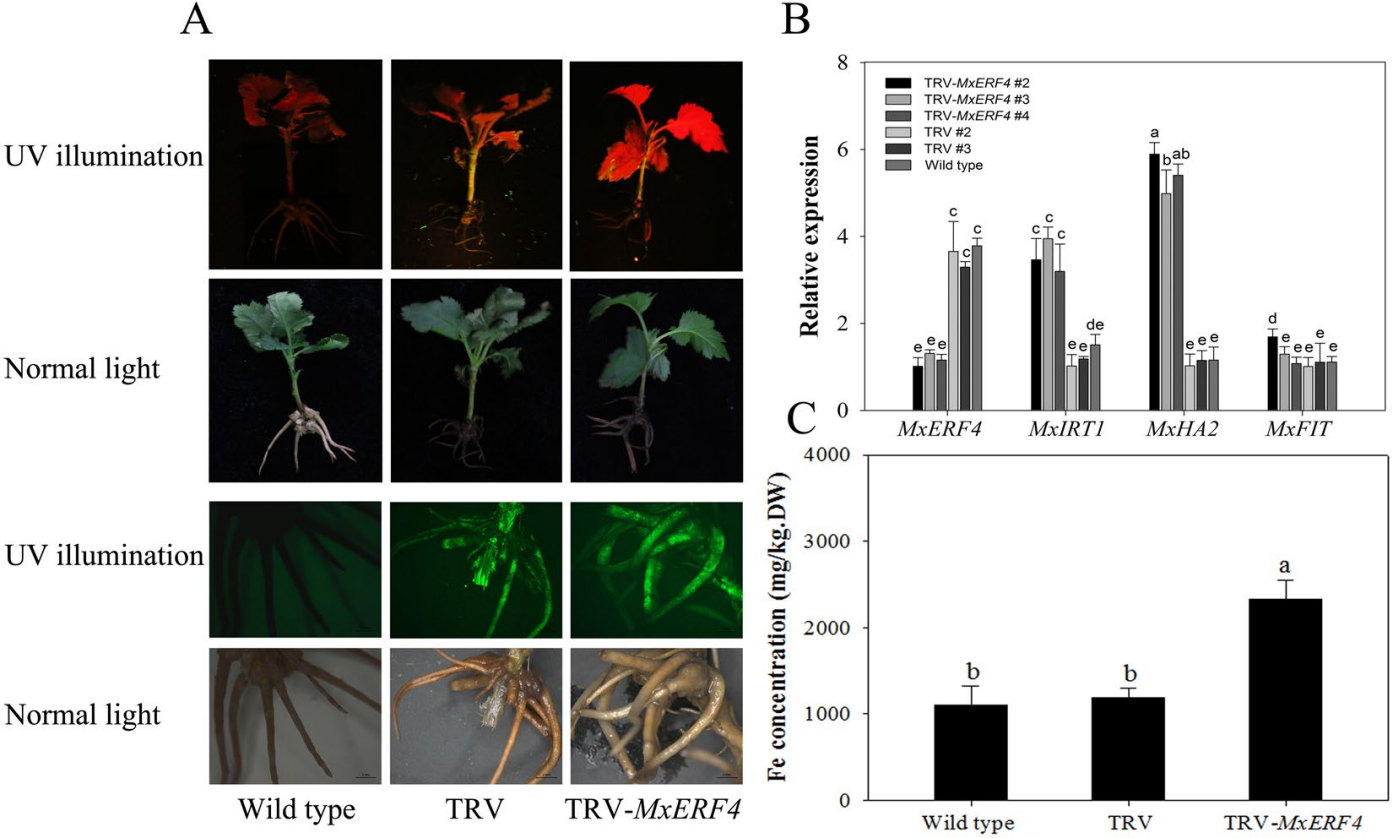
Transient expression of *MxERF4* in *Malus xiaojinensis*. The *M. xiaojinensis* seedlings were infiltrated with agrobacterium containing a TRV control (pTRV1 + pTRV2-GFP), or TRV carrying a *MxERF4* fragment (pTRV1 + pTRV2-GFP-*MxERF4*). (**A**) Green fluorescent protein (GFP) imaging of TRV-GFP-*MxERF4* infiltrated *M. xiaojinensis* seedlings by UV and fluorescence microscopy. (**B**) Quantitative real-time reverse transcription-PCR (qRT-PCR) analysis of *MxIRT1, MxHA2* and *MxFIT* expression in silenced roots. Values are means ± SE of three replicates. Different letters represent statistically different means at P < 0.05 (one-way ANOVA analysis with Duncan post-hoc test). (**C**) Analysis of Fe concentration in silenced roots. Bars represent means ± SE of three replicates. Different letters represent statistically different means at P < 0.05 (one-way ANOVA analysis with Duncan post-hoc test).

**Figure 8:**
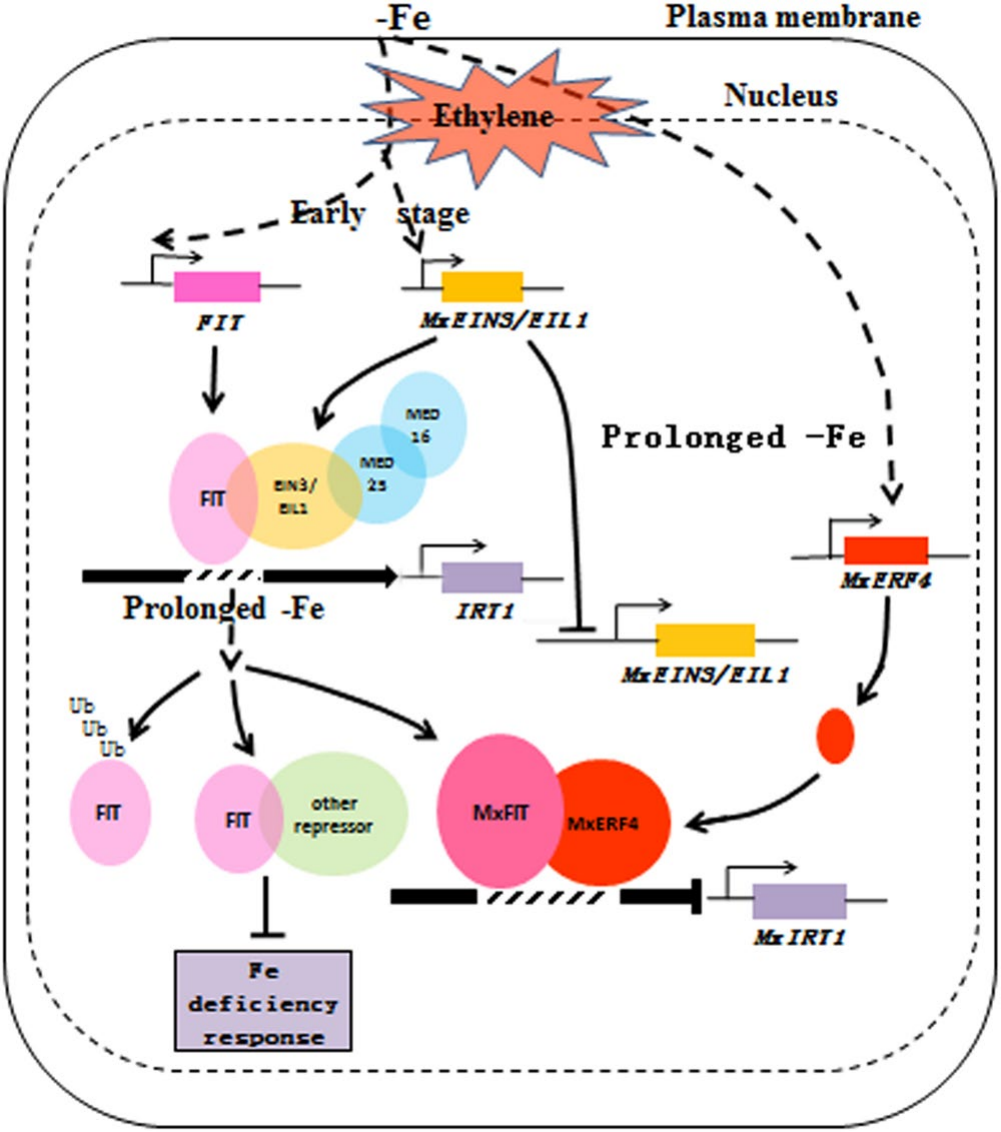
Model of MxERF4 action in suppressing Fe uptake. The early stages of Fe deficiency leads to the induced expression of MxEIN3/EIL1. After prolonged Fe starvation, the expression of MxEIN3/EIL1 is inhibited and the expression of MxERF4 is up-regulated. We propose that MxERF4 can act as a transcriptional repressor through its EAR motif. Ethylene causes an enhanced Fe deficiency adaptive response at the early stages of the response by up-regulating EIN3/EIL1 and the MED complex, but the ethylene response slows down once it has been initiated via the action of MxERF4. MxERF4 eventually interact with MxFIT and suppresses *MxIRT1* gene expression.

To further test the function of MxERF4 as a regulator, we used virus induced gene silencing (VIGS) to suppress the expression of *MxERF4* in *M. xiaojinensis* plantlets. *M. xiaojinensis* tissue culture plantlets infiltrated with the virus containing the TRV-GFP*-MxERF4* (TRV, for silencing) (Fig. 7), and green fluorescent protein (GFP) fluorescence was observed in leaves, stems and new roots (Fig. 7A), indicating that the virus had spread through the plants. Suppression of *MxERF4* expression, using a TRV-GFP*-MxERF4* plasmid containing a partial *MxERF4* ORF, caused an increase in *MxIRT1* and *MxHA2* expression; however only minor changes in *MxFIT* expression were observed (Fig. 7B). And the Fe content in silenced roots (virus containing the TRV-GFP-*MxERF4*) was higher than wild type and empty vector (Fig. 7B). The results of this study were used to generate a model of the regulatory system involving MxERF4. Exposure of *M. xiaojinensis* plants to Fe deficiency, induces ethylene production, and while ethylene sensitivity increases in the earlier stages of Fe deficiency it subsequently decreases. MxERF4 act as an MxFIT interacting partner, and the protein complex binds to the *MxIRT1* promoter, thereby suppresses *MxIRT1* expression which results in a reduction in Fe uptake. In this model, the ethylene response can be slowed down once it has been initiated, and this dampening process may require a transient inhibition of Fe acquisition via MxERF4 action (Fig. 8).

## Discussion

It has long been known that Fe deficiency can induce a wide range of adaptive responses, but the existence of a dampening mechanism associated with these responses has been unclear, particularly in woody plants^38^. We show here that MxERF4 suppresses Fe uptake by binding to the *MxIRT1* promoter and, importantly, that a key factor in modulating the ethylene response is a counterbalanced Fe uptake response.

In different dicotyledonous plants, experiments indicated a physiological connection between iron deficiency signaling and ethylene, in that ethylene production increases with Fe deficiency treatment^7,46–48^. Applying ethylene precursors to plants promotes Fe deficiency responses, such as the activation of *IRT1* and *FRO2* gene expression^10,48,49^. Our data suggest that exogenous application of the ethylene inhibitor, AVG, suppresses the Fe deficiency response, since it prevented Fe deficiency-induced ethylene accumulation and concomitantly blocked the up-regulation of Fe(III) reductase activity and the root proton extrusion, while addition of ethephon (ACP) reversed these physiological responses (Fig. 2). However, once the ethylene signal has been initiated, the mechanism by which the ethylene response can counteract prolonged Fe treatment is unknown.

FIT can interact with transcription factors, such as basic helix-loop-helix (bHLH) proteins^50,51^, the EIN3 and EIL1^23^and the mediator subunit MED16^25^, which play a central role that governs Fe deficiency responses. And all these interactions were shown to promote FIT activity^23,25,50,51^. Conversely, ZAT12 from *Arabidopsis thaliana*, was reported to suppress Fe acquisition^21^. In our study, the identification of MxERF4 as a suppressor of *MxIRT1* expression provides insights into counterbalanced effects at the molecular level. Most of the ERF proteins characterized to date have been shown to act as transcriptional activators^28–33,52–54^; however, some ERF proteins with EAR motif can function as transcriptional repressors, although little is known about their physiological roles. From our previously published transcriptional profile data^44^, we identified an EAR motif ERF (MxERF4) as a regulator capable of modulating ethylene response to counterbalance the Fe response. Using VIGS, in combination with an activity repression analysis, we speculated that MxERF4 directly binds to the *MxIRT1* promoter and that the EAR motif of MxERF4 was required for this suppression. Upon Fe deficiency, *MxERF4* expression levels was inhibited during the first 6 days, but was induced at day 9. With prolonged Fe deficiency treatment, *MxERF4* could be served as a negative regulator. In the later Fe deficiency stage, in order to prevent damage from any over accumulation of other metals, downregulation of the metal uptake machinery is required, which are transported by *IRT1*^55^. It may be that the suppression ability of MxERF4 binding to *MxIRT1* promoter.

*Arabidopsis*, FIT is not able to activate *IRT1* promoter on its own but only in combination with another bHLH of the Ib subgroup^50,51^. Compared with model plant *Arabidopsis*, woody plants have high level of genetic variation; the use of highly heterozygous woody plant ‘*Malus xiaojinensis*’ has allowed us to demonstrate that FIT can activate *IRT1* promoter from our results. Also our experiment showed the interaction of MxIRO2/MxFIT with the *MxIRT1* promoter enhanced the activity of GUS (Fig. S2). This regulation will be the target of future studies.

Several plant signaling molecules are known to modulate the Fe deficiency response, either by inducing it, such as nitric oxide^10,56–58^ and auxin^38,58,59^, or by suppressing it, as is the case with cytokinin^60^. Ethylene and auxin have been shown which can regulate iron-deficiency responses from the pharmacological experiments and use of some plant mutants^6,7,38,58,59,61^. Application of ethylene precursor or auxin mimicked iron deficiency in inducing the iron adaptive responses^6–8,61^. Furthermore, our research showed that IAA plays a key role to mediate the iron deficiency responses in *M. xiaojinensis*^38^. Blum *et al*. showed the balance between the ethylene and auxin leads to an almost continuous *IRT1* presence under iron deficiency condition^62^. Dominant ethylene presence induces *IRT1* expression in the root with iron deficient treatment^62^. Thus, we proposed that both ethylene and auxin play crucial characters in iron deficiency responses and the cross-talks among ethylene and auxin need to be further explored.

Previous studies have suggested a molecular link between Fe deficiency responses and hormone signaling regulation, and we report here a suppression mechanism that dampens the plant responses. This model provides a basic molecular framework for future investigation of the regulatory systems that allow environmental adaption.

## Materials and Methods

### Plant material and growth conditions

*Malus xiaojinensis* seedlings were cultivated until stem lignification in Murashige and Skoog (MS) medium with 0.3 mg/L indolebutyric acid (IBA) + 0.3 mg/L 6-Benzyl Aminopurine (6-BA), and were then transferred to rooting medium (1/2 MS + 0.5 mg/L IBA) for a month, until white roots were visible. The seedlings were removed from the culture medium and washed with distilled water three times to remove the agar. After pretreatment, the seedlings were cultivated in a standard nutrition solution^58^, with the pH adjusted to 6.0 using 1 M NaOH. The nutrition solution was replaced every week. Plants were grown for 60 d in a growth room at 25 ± 2 °C day/17 ± 2 °C night with a 16 h photoperiod at a light intensity of 250 µmol·m^−2^·s^−1^. After a variable precultivation period, some plants were transferred to nutrient solutions representing Fe-deficient (0 µM Fe), Fe-deficient with added ethylene inhibitor (0 µM Fe + 10 µM AVG), Fe-sufficient (40 µM Fe) and Fe-sufficient with added ethephon (40 µM Fe + 100 mg/L ACP) conditions. For RNA extraction, roots were harvested after 0, 2, 6 and 9 d of the above treatments.

### Isolation of total RNA and quantitative real-time reverse transcription-PCR

Total RNA was extracted from the roots using a modified CTAB method^63^ and treated with DNase I to remove DNA contamination (TaKaRa Biotechnology Co., Ltd., Dalian, China). To generate cDNA, the RNA samples were reverse-transcribed using an oligo-dT primer and reverse transcriptase (TaKaRa Biotechnology Co., Ltd., Dalian, China), according to the manufacturer’s instructions. The relative expression levels of genes were detected using an Applied Biosystems 7500 real-time PCR system. The housekeeping gene β-actin which is degenerate primer (MDP0000896590, MDP0000428264 and MDP0000170174) was used as the control. The primers were designed using the Primer Premier 5 software (Premier Biosoft, USA)^64^. The efficacy of the primers was confirmed by qRT-PCR. Amplifications using these primers yielded 100–200 bp products, which were subjected to melting curve analyses. The relative expression was calculated according to the 2^−ΔΔCT^ method^65^. Primers are listed in Supplemental Table S1. Each reaction was performed in triplicate.

### Chlorophyll content analysis

Leaf samples (0.2 g) were collected and transferred to 80% acetone for 24 h. Chlorophyll content was detected by measuring the absorbance of the solution at A_645_ and A_663_ using a spectrophotometer^66^.

### Fe content determination

Samples were collected and dried at 105 °C for 20 min, followed by 70 °C for 6 days, and separately ground to a powder using grinding rods. Subsequently, 0.1 g of the powder was transferred to 10 mL 1 M HCl and the samples were shaken at 180 rpm for 5 h. The Fe content was measured by atomic absorption spectrometry after filtration (Z-5000, Hitachi, Tokyo, Japan)^67,68^.

### Measurements of net H^+^ fluxes with non-invasive microtest technique (NMT)

Net H^+^ fluxes were measured using a BIO-IM Series NMT system (Younger USA) at the Xuyue Beijing NMT Research Service Center, China. H^+^ ion-selective microelectrodes were placed 2 mm from the root cap and the samples were placed in the testing solution^69^ at pH 6.0. Only electrodes with a Nernstian slope >50 mV/decade were used in this study. H^+^ fluxes were calculated using the JCal V3.3 (a free MS Excel spreadsheet, youngerusa.com or xuyue.net). Ion flux was calculated by Fick’s law of diffusion, which demonstrated in the NMT Experiments instruction in xuyue.net.

### Measurement of Fe^3+^ reductase activity and rhizosphere pH

The solution pH of different treatments (0 µM Fe, 0 µM Fe + 10 µM AVG, 40 µM Fe and 40 µM Fe + 100 mg/L ACP) was original adjusted to 6.3, whereafter the pH of the solutions was measured when the plants were incubated in the solution for 1.5 month. Root Fe^3+^ reductase activity was determined as described by Schikora and Schmidt^59^ and Li *et al*.^70^, with some modifications. The plant roots were soaked in a saturated CaSO_4_ solution for 5 minutes, washed with distilled water, and the plants were then transferred to the nutrient solution described above, with the addition of Fe-EDTA (0.1 mM) and 2,2-bipyridyl (0.4 mM), then incubated in the dark for 2 h. The environmental conditions during the measurements were the same as those for normal growth. The reducing capacity of the solution was determined by measuring the concentration of the Fe^2+^-dipyidyl complex formed at A_520_ using a spectrophotometer (Unico UV-2012). Each experiment was repeated at least three times. For some treatments, the location of the ferric reductase activity along the roots was visualized on agar (0.7%, w/v) plates with a ferric reduction assay solution consisting of Fe-EDTA (0.5 mM) and FerroZine (0.5 mM).

### Yeast one hybrid (Y1H) and two hybrid (Y2H) assays

The CDS of MxERF4 and MxFIT were ligated into the pJG4-5 vector, *MxIRT1* promoter was cloned into the pLacZi vector which contains *LacZ* reporter gene^71^. And the Y1H assay was conducted as described in the Yeast Protocols Handbook (Clontech). Transformants were grown on a minimal medium/-Ura,-Trp containing X-gal (5-bromo-4–chloro-3–indolyl-β–D–galactopyranoside) to observe the color development of yeast colonies. Vector construction primers are listed in Supplemental Table S1.

For the Y2H assay, full-length cDNAs of MxERF4 and MxFIT were cloned into the pGBKT7 and pGADT7 vectors from Clontech. All constructs were transformed into yeast (*S. cerevisiae*) strain AH109 using the lithium acetate method, and yeast cells were grown on a minimal medium/-Leu,-Trp according to the manufacturer’s instructions (Clontech). Transformed colonies were plated onto a minimal medium/-Leu,-Trp,-His,-adenine containing 20 mg/mL X-gal to test for possible interactions. Primers used for vector construction are listed in Supplemental Table S1.

For the yeast transcription activation assay, full-length cDNAs of MxERF4 and the MxERF4 EAR sequence were separately cloned into a strong transcriptional activation vector pBD-GAL4-VP16^28^. Constructs were transformed into yeast (*S. cerevisiae*) strain AH109 using the lithium acetate method, as above, and yeast cells were grown on a minimal medium/-Trp according to the manufacturer’s instructions (Clontech). Transformed colonies were plated onto a minimal medium/-Trp,-His containing 20 mg/mL X-gal and ortho-nitrophenyl-β-D-galactopyranoside (ONPG) as substrates. Primers used for vector construction are listed in Supplemental Table S1. Experiments were repeated three times.

### BiFC Assays

To generate the BiFC constructs, the full-length cDNA sequences of MxERF4 and MxFIT were cloned into the pSPYNE-35S and pSPYCE-35S vectors^72^. The primers used for the BiFC assays are listed in Supplemental Table S1. Coexpression was observed in tobacco (*Nicotiana tabacum*) leaves as described by Schütze *et al*.^45^. The fluorescence of the fusion proteins was detected 3 days after Agrobacterium infiltration. Fluorescence images were acquired using a Nikon D-ECLIPSE C1 spectral confocal laser-scanning system. YFP and brightfield images were generated by excitation at 488 and 543 nm, respectively. A Nikon ECLIPSE TE2000-E Inverted fluorescence microscope was used for the fluorescence analysis.

### Transient transformation analysis in tobacco leaf

The full-length coding regions (CDS) of MxERF4 and MxFIT were cloned into the KpnI and SalI sites of the pCAMBIA2300 vector to form the pCAMBIA2300- *MxERF4* and pCAMBIA2300-*MxFIT* constructs. The pCAMBIA1301 plasmid was used as normalization control which contains the GUS reporter driven by the 35S promoter, the promoter of *MxIRT1* was fused into pCAMBIA1301 and replaced the 35S promoter. Then the four groups (pCAMBIA2300-*MxFIT/MxERF4* + pCAMBIA1301-*proMxIRT1*; pCAMBIA2300-*MxFIT* + pCAMBIA1301-*proMxIRT1*; pCAMBIA2300-*MxERF4* + pCAMBIA1301-*proMxIRT1*; pCAMBIA1301-*proMxIRT1*) were respectively infiltrated tobacco (*Nicotiana tabacum*) leaves, tobacco plants were grown for 2 d after infiltration, and then collected the transformed leaves soaked in GUS staining solution at 37 °C. Total removal of chlorophyll using 70% alcohol decolorization, and observed the expression of GUS.

### Silencing of *MxERF4* in *M. xiaojinensis* by VIGS

Silencing of *MxERF4* expression by virus induced gene silencing (VIGS) was performed as described by Dai *et al*.^73^, with some modifications. A 300-bp fragment at the 3′ UTR of *MxERF4* was amplified using a forward primer with an *Eco*RI restriction site and a reverse primer with a *Xba*I restriction site. The pTRV1 and pTRV2-GFP vector from Professor Gao and 3′ UTR of *MxERF4* cloned into pTRV2-GFP vector (pTRV2-GFP-*MxERF4*). The vectors of pTRV1, pTRV2-GFP and pTRV2-GFP-*MxERF4* were transformed individually into *Agrobacterium tumefaciens* strain GV3101, and the transformed *A. tumefaciens* lines were cultured for 24 h in Luria–Bertani medium^55^ supplemented with 50 mg/ml kanamycin and 50 mg/ml rifampicin. The cultures were harvested, and suspended in infiltration buffer (10 mM MgCl_2_, 1.5 mM acetosyringone, 10 M MES, pH 5.6) to a final OD_600_ of approximately 1.0. Mixtures of cultures containing an equal ratio (v/v) of pTRV1 and pTRV2-GFP (control), pTRV1 and pTRV2-GFP-*MxERF4* were placed at room temperature in the dark for 3–4 h before vacuum infiltration. Tissue culture seedlings were collected and vacuum infiltration was performed by immersing them in the bacterial suspension solution and infiltrating under a vacuum at 0.7 MPa. After release of the vacuum, seedlings were washed in deionized water and kept in deionized water for 3 days at 8 °C, followed by cultivation in a standard nutrition solution^67^. The nutrition solution was replaced every week. Plants were grown in a growth room at 25 ± 2 °C day/17 ± 2 °C night with a 16 h photoperiod at a light intensity of 250 µmol·m^−2^·s^−1^. After 12 d, fluorescence in the transformed plants was observed using a fluorescence microscope (ZEISS), and the roots were used for RNA isolation.

### Statistical analysis

The Statistical Product and Service Solutions (SPSS) software (IBM Co, Armonk, USA) was used for statistical analysis. All experimental data were tested using one-way analysis of variance (ANOVA) and Duncan’s multiple-range test.

## Electronic supplementary material

Supplementary Data
