# Supplementary material for: An ethylene response factor (MxERF4) functions as a repressor of Fe acquisition in Malus xiaojinensis
Source: Sci Rep. 2018 Jan 18;8:1068. doi: 10.1038/s41598-018-19518-4 (PMC5773544; doi:10.1038/s41598-018-19518-4)
Supplement: Supplementary file 1 — Supplementary Data [file 41598_2018_19518_MOESM1_ESM.doc]

**An ethylene response factor (*MxERF4*) functions as** **a negative regulator of Fe acquisition in *Malus xiaojinensis***

**Wei Liu^1,2*^, Ting Wu^1,2*^, Qiwei Li^1,2^**, **Xinzhong Zhang^1,2^, Xuefeng Xu^1,2^, Tianhong Li^2^, Zhenhai Han^1^****^,2^ and Yi Wang^1,2†^**

^1^Institute for Horticultural Plants, China Agricultural University, Beijing 100193, P. R. China

^2^ Key Laboratory of Physiology and Molecular Biology of Tree Fruit of Beijing, China Agricultural University, Beijing 100193, P. R. China

**Footnote:**

**^*^**These authors contributed equally to this work.

^†^To whom correspondence should be addressed.

Yi Wang, Email: wangyi@cau.edu.cn

Zhenhai Han，Email：rschan@cau.edu.cn

**
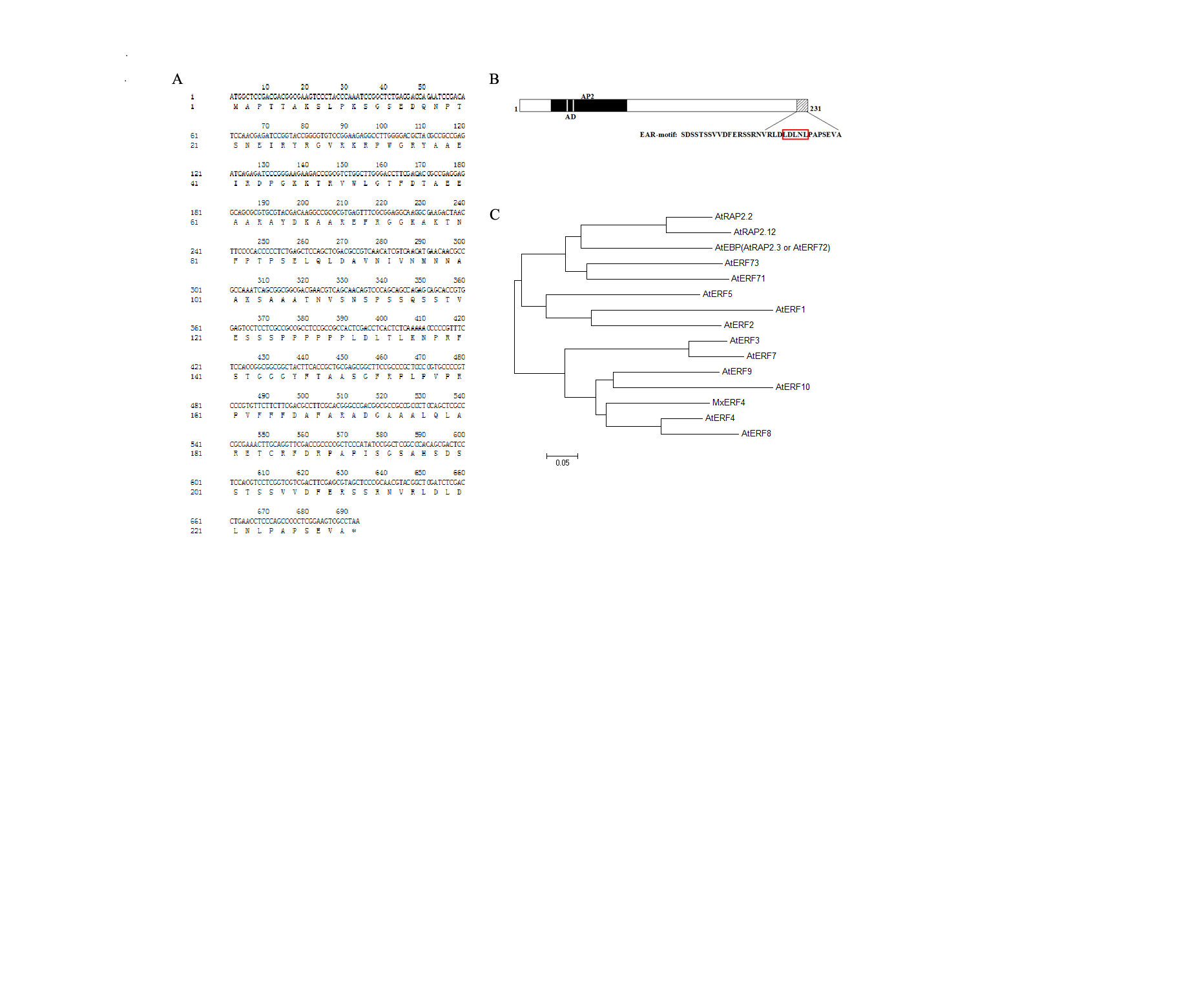
**

**Figure S1.** **Sequence and phylogenetic tree analysis of *MxERF4*. (A)** The CDS seguence and protein sequence of MxERF4. **(B)** The EAR-motif of MxERF4 is located in C-terminus of amino acid. **(C)** Phylogenetic analysis of homologous ERFs in the apple and *A. thaliana* genomes.


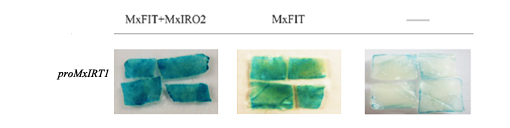


**Figure S2.** Effect of transcription factors on the activity of *IRT1* promoter.

**Table S1.** Primer sequences used in this study.

| **Gene**  **name** | **ID** | **Sequence (5’-3’)** | **Used for** |
| --- | --- | --- | --- |
|  |  |  |  |
| MxERF4 | MxERF4-F1 | ATGGCTCCGACGACGGCGAA | Gene amplification |
|  | MxERF4-R1 | TTAGGCGACTTCCGAGGGGGCTG |  |
|  | MxERF4-F2 | CGGAATTC ACTCCGGCCGCCCGCCGCGAAA | VIGS |
|  | MxERF4-R2 | GCTCTAGA AACAGAGAGAAAAGTAAGATT |  |
|  | MxERF4-F3 | CGGAATTC ATGGCTCCGACGACGGCGAA | Yeast one-hybrid |
|  | MxERF4-R3 | CCGCTCGAG TTAGGCGACTTCCGAGGGGGCTG |  |
|  | MxERF4-F4/5 | CGGAATTC ATGGCTCCGACGACGGCGAA | Yeast two-hybrid |
|  | MxERF4-R4/5 | CGGGATCC TTAGGCGACTTCCGAGGGGGCTG |  |
|  | MxERF4-F6/7 | GCTCTAGA ATGGCTCCGACGACGGCGAA | BiFC |
|  | MxERF4-R6/7 | CGGGATCC GGCGACTTCCGAGGGGGCTG |  |
|  | MxEAR-F | CGGGATCC TCCACGTCCTCGGTCGTCGA | Yeast transcription activation |
|  | MxEAR-R | AACTGCAG GGCGACTTCCGAGGGGGCTG |  |
| MxFIT | MxFIT-F1 | CGGGATCC ATGGATTCGCTGGGAAACCA | Yeast two-hybrid |
|  | MxFIT-R1 | CCGCTCGAG TTAGGCTGAATCCAGAAGC |  |
|  | MxFIT-F2/3 | GCTCTAGA ATGGATTCGCTGGGAAACCA | BiFC |
|  | MxFIT-R2/3 | CGGGATCC GGCTGAGAATCCAGAAGC |  |
| MxIRT1 | ProMxIRT1-F1 | CGGAATTC GCCTGATATGAGCTGCTTC | Promoter amplification |
|  | ProMxIRT1-R1 | CCGCTCGAG TGACCCTGATTTTTAGCTC |  |
| MxERF4 | MxERF4-F8 | TGTTCTTCTTCGACGCCTTC | qRT-PCR |
|  | MxERF4-R8 | AGGGAGGTTCAGGTCGAGAT |  |
| MxEIN3 | MxEIN3-F | CATTGCTCAACCACCAACAC | qRT-PCR |
|  | MxEIN3-R | TTTGACTGGTTCACCTGCTG |  |
| MxEIL1 | MxEIL1-F | TCCCTGGGAAGAATGAACAC | qRT-PCR |
|  | MxEIL1-R | TTACCAGTAGGCCACCAAGG |  |
| MxFIT | MxFIT-F | GGGAAACCATCAAGGAGGTCATA | qRT-PCR |
|  | MxFIT-R | AGCCATTCATCATAAGGTCAGGA |  |
| MxHA2 | MxHA2-F | ATCTGCCCTTACTGGGGAGT | qRT-PCR |
|  | MxHA2-R | AAATGCAGAAGTTCCCGATG |  |
| MxIRT1 | MxIRT1-F | CATGGCACAAGTTCCCTTTT | qRT-PCR |
|  | MxIRT1-R | ACCACAGCCATCTCTTGGTC |  |
| MxActin2 | MxActin2-F | TGGTGAGGCTCTATTCCAAC | qRT-PCR |
|  | MxActin2-R | TGGCATATACTCTGGAGGCT |  |
